# Buried Bumper Syndrome Revisited: A Rare but Potentially Fatal Complication of PEG Tube Placement

**DOI:** 10.1155/2014/634953

**Published:** 2014-01-16

**Authors:** Saptarshi Biswas, Sujana Dontukurthy, Mathew G. Rosenzweig, Ravi Kothuru, Sunil Abrol

**Affiliations:** Department of General Surgery, Brookdale University Hospital Medical Center, Brooklyn, NY 11212, USA

## Abstract

Percutaneous endoscopic gastrostomy (PEG) has been used for providing enteral access to patients who require long-term enteral nutrition for years. Although generally considered safe, PEG tube placement can be associated with many immediate and delayed complications. Buried bumper syndrome (BBS) is one of the uncommon and late complications of percutaneous endoscopic gastrostomy (PEG) placement. It occurs when the internal bumper of the PEG tube erodes into the gastric wall and lodges itself between the gastric wall and skin. This can lead to a variety of additional complications such as wound infection, peritonitis, and necrotizing fasciitis. We present here a case of buried bumper syndrome which caused extensive necrosis of the anterior abdominal wall.

## 1. Introduction

Percutaneous endoscopic gastrostomy (PEG) was first reported in the literature in 1980 as an alternative way to provide tube feeding for patients without a laparotomy [1]. Today, PEG placement is widely accepted as a safe technique to provide long-term enteral nutrition for a variety of patients including those with neurologic deficits and swallowing disorders and those with oropharyngeal or esophageal tumors and various hypercatabolic states like burns, short bowel syndrome, and major traumas [2]. Although considered a safe procedure, immediate and delayed complications have been described with the PEG placement. These complications vary from minor complications like wound infections to major life threatening complications like peritonitis and buried bumper syndrome. BBS is an uncommon but serious complication of PEG, occurring in 0.3–2–4% of patients [3]. We present here a case of BBS followed by a discussion of its etiology, management, and prevention.

## 2. Case description

A 70-year-old female with multiple comorbidities presented to the ER from the nursing home with symptoms suggestive of septic shock. At the time of admission, the patient was undergoing active treatment for urinary tract infection in the nursing home. Physical examination of the patient revealed respiratory distress and hypotension, so emergency intubation was done and vasopressors started to maintain blood pressure. Empiric broad spectrum antibiotics were initiated for septic shock. Patient was then transferred to the medical intensive care unit for further management.

Patient history revealed that the PEG tube was inserted one year prior due to dysphagia from a stroke. Upon abdominal examination, the PEG tube was in place in the epigastric area with signs of edema and erythema on the right lateral side of the abdomen.

**Figure 1:**
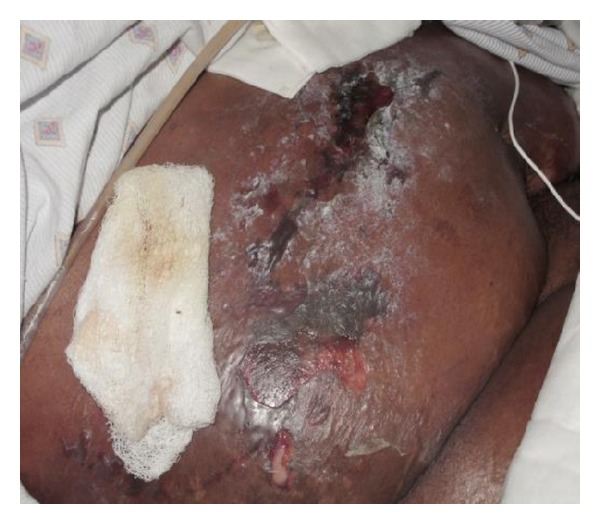
Anterior abdominal wall showing edema, erythema, and ruptured bullae over the abdomen.

Bullae were spread diffusely across the abdomen (Figure 1), and gastric contents were noted to be leaking around the PEG tube. The patient localized tenderness to palpation, and bowel sounds were normal with no rebound or guarding.

The general surgery team was consulted for PEG tube position and abdominal wall erythema and edema.

Laboratory studies revealed leukocytosis of 18000 cells/cubic mm, hemoglobin of 5.3 g/dl, hematocrit of 15.2%, and an INR greater than 10 as the patient was on regular Coumadin for chronic atrial fibrillation. Computed tomography scan of the abdomen and pelvis was recommended to confirm PEG tube position and to evaluate for retroperitoneal hematoma in view of high INR and low hemoglobin. The nursing staff was subsequently instructed to hold feeding through the PEG tube till its position could be confirmed with the CT scan.

**Figure 2:**
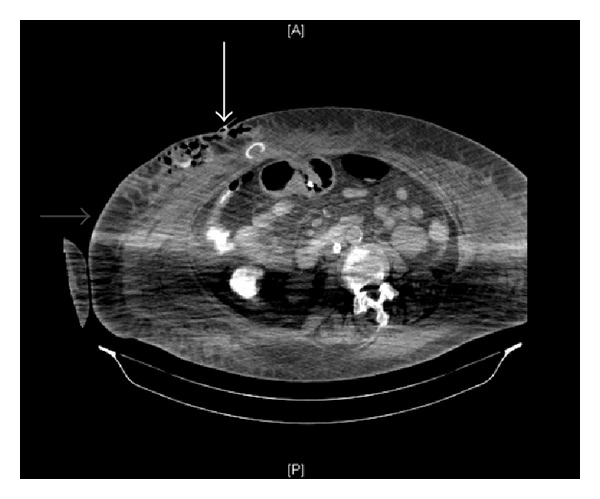
CT image of the abdomen. (1) The solid arrow indicates dislodgement of the internal bumper of the PEG tube into the abdominal wall outside the peritoneum. (2) The hollow arrow shows subcutaneous collection of fluid and air in the abdominal wall.

**Figure 3:**
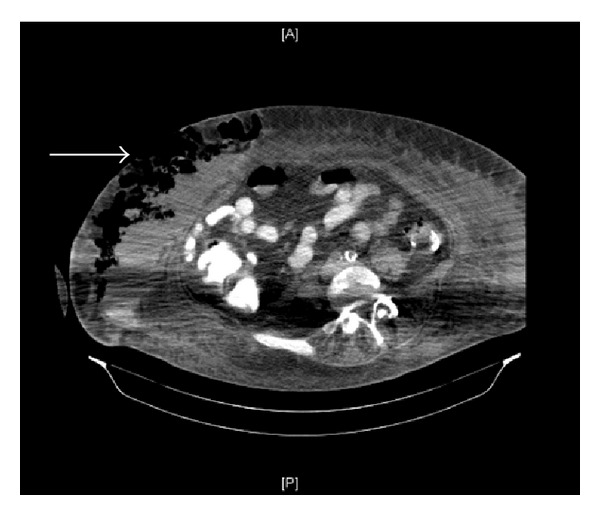
CT image of the abdomen. The arrow indicates extensive subcutaneous collection of fluid and air in the abdominal wall.

CT scan of the abdomen showed dislodgement of the internal button of the gastrostomy tube into the abdominal wall and a large collection measuring 10 × 7.5 × 20 cm. The collection showed equal parts of gas and fluid density in the subcutaneous compartment of the right anterolateral abdominal wall just lateral to the percutaneous gastrostomy tube outside the muscle and peritoneal reflection (Figures 2 and 3).

After explaining the benefits and risks of the surgical procedure for drainage in the operating room, the intervention was denied by the patient's next of kin. Aspiration of the subcutaneous collection by the interventional radiologist was scheduled; however, the plan was withheld due to hemodynamic instability and the risk of transport to the radiology suite. A plan was made for bedside incision, drainage, and debridement of the subcutaneous collection. Vitamin K and FFP was administered for increased INR. After explaining the risks and benefits, informed consent was obtained from the patient's next of kin.

**Figure 4:**
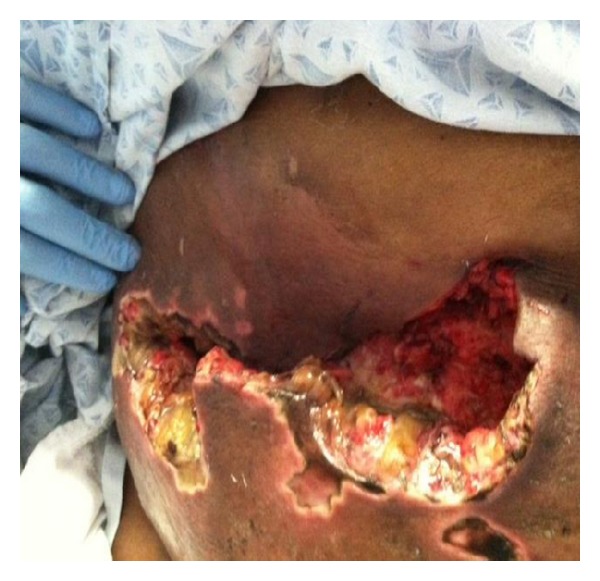
Anterior abdominal wall with erythema and edema prior to debridement.

**Figure 5:**
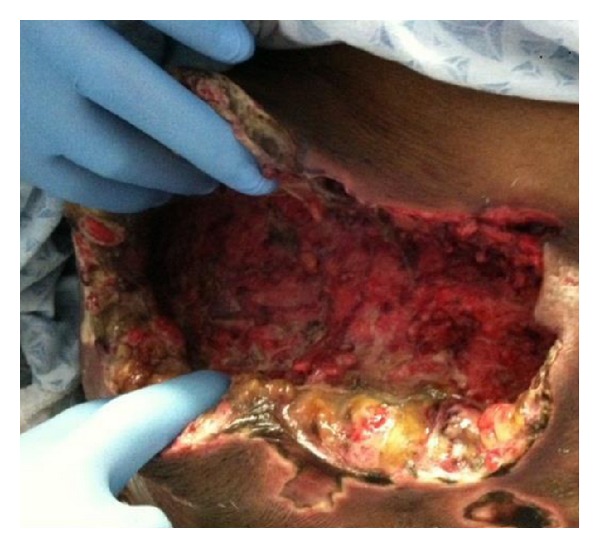
Bedside debridement and pulse lavage of the subcutaneous collection of the anterior abdominal wall.

**Figure 6:**
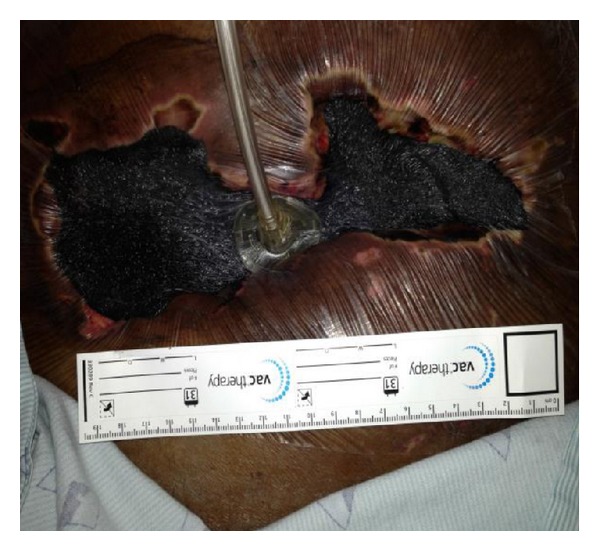
Wound vac after debridement for further drainage.

Bedside debridement was performed, and over 600 milliliters of foul smelling brownish fluid was aspirated from the wound (Figures 4 and 5). The fluid and the PEG tube tip were sent for culture and sensitivity. Wound vacuum was inserted and kept in situ for further drainage (Figure 6). The aspirated fluid and PEG tube tip culture and sensitivity revealed Klebsiella Pneumonia and Candida Vulgaris. Despite resuscitative efforts, the patient expired 10 days after debridement from septic shock.

Wound vac was inserted after bedside debridement and drainage. Appropriate antibiotics and antifungals were initiated according to the microbial sensitivity.

## 3. Discussion

PEG placement complications can be minor ranging from wound infection around the PEG tube to major complications like BBS, necrotizing fasciitis, and colocutaneous fistula. The overall complication rate ranges from 4% to 23.8% of cases [4–7]. Three to 4% of all cases are affected by major complications [4, 6, 8]. The more common minor complications occur between 7.4% and 20.0% of cases [4, 6, 8]. Generally, complications are more likely to occur with elderly patients, especially those with comorbid conditions, as well as those with a past history of aspiration [9]. BBS, first described in 1988, is considered a late and rare complication of PEG placement [10]. It occurs when the internal bumper of the feeding tube erodes into the gastric wall leading to ischemic necrosis and the ultimate migration of the internal bumper and lodging itself between the gastric wall and the skin. A relationship is believed to exist between tightening of the external bolster in an effort to prevent leaking of gastric contents causing increased tension in the tube [3]. Other contributing factors include gastric acid alteration of the internal bumper, PEG tube characteristics such as a hard plastic composition, and inadequate patient care [11].

Although many risk factors like obesity, rapid weight gain, patient manipulation, gauze placement beneath the external bumper instead of over it, chronic cough, tube manipulation by inexperienced personnel, and malnutrition have been associated with BBS, obesity is considered as the single most important risk factor for this syndrome [3]. It can be ascertained that any unnecessary increased tension of the tube can lead to BBS over a period of time. While the earliest reported complication occurred at 8 days after insertion, in a range of 1–50 months the majority of BBS occur with a median of 18 months [9, 12]. Ultimately, the migration of the internal bolster can lead to a loss of feeding access and a variety of other minor and major complications as previously discussed. Patients with this syndrome typically present with leakage around the PEG tube or signs of infection like edema or erythema, an immobile catheter, and abdominal pain or resistance to administer formula infusion. Diagnosis of BBS is made clinically and confirmed endoscopically or with computed tomography [3].

The mainstay of treatment for these patients includes the removal of the buried bumper, even in the asymptomatic patient in order to avoid further complications such as stomach perforation, peritonitis, and infection of the subcutaneous tissue [11]. Various internal techniques including surgery or endoscopic snare retrieval through the mouth can be implemented for tube removal [11, 13]. Often times, simple external traction is possible with a collapsible internal bumper [2]. Additional techniques are currently being described such as using an angioplasty balloon dilator under radiological guidance to avoid surgery [14].

While the current literature lacks strong evidence to support a specific preventive practice, possible considerations have been suggested. Among these are allowing for an additional 1.5–2 cm between the external bumper and the skin, gently rotating and manipulating the PEG in and out daily, and measuring the length of the external portion of the tube in order to recognize migration and avoid unnecessary tube traction [3, 11].

## 4. Conclusion

BBS is a rare and typically late presenting complication of PEG tube placement. Early recognition of this complication reduces the life threatening consequences involved. A multidisciplinary team approach and patient education are essential efforts for preventing BBS.
